# Integrating Proximal and Remote Sensing with Machine Learning for Pasture Biomass Estimation

**DOI:** 10.3390/s25071987

**Published:** 2025-03-22

**Authors:** Bernardo Cândido, Ushasree Mindala, Hamid Ebrahimy, Zhou Zhang, Robert Kallenbach

**Affiliations:** 1 Division of Plant Science and Technology, University of Missouri, Columbia, MO 65201, USA; ummbv@missouri.edu (U.M.); kallenbachr@missouri.edu (R.K.); 2 Department of Biological Systems Engineering, University of Wisconsin-Madison, Madison, WI 53706, USA; hamdebrahimy@gmail.com (H.E.); zzhang347@wisc.edu (Z.Z.)

**Keywords:** pasture biomass, proximal sensing, remote sensing, vegetation indices, machine learning

## Abstract

This study tackles the challenge of accurately estimating pasture biomass by integrating proximal sensing, remote sensing, and machine learning techniques. Field measurements of vegetation height collected using the PaddockTrac ultrasonic sensor were combined with vegetation indices (e.g., NDVI, MSAVI2) derived from Landsat 7 and Sentinel-2 satellite data. We applied the Boruta algorithm for feature selection to identify influential biophysical predictors and evaluated four machine learning models—Linear Regression, Decision Tree, Random Forest, and XGBoost—for biomass prediction. XGBoost consistently performed the best, achieving an R^2^ of 0.86, an MAE of 414 kg ha⁻^1^, and an RMSE of 538 kg ha⁻^1^ using Landsat 7 data across multiple years. Sentinel-2’s red-edge indices did not substantially improve predictions, suggesting a limited benefit from finer spectral resolutions in this homogenous pasture context. Nonetheless, these indices may offer value in more complex vegetation scenarios. The findings emphasize the effectiveness of combining detailed ground-based measurements with advanced machine learning and remote sensing data, providing a scalable and accurate approach to biomass estimation. This integrated framework provides practical insights for precision agriculture and optimized pasture management, significantly advancing efficient and sustainable rangeland monitoring.

## 1. Introduction

Accurate pasture biomass estimation is crucial for sustainable management, optimizing livestock production, and monitoring ecosystem health. It enables informed decision-making about grazing strategies and carrying capacity. While traditional methods like manual clipping provide reliable data, they are labor-intensive and impractical for large-scale applications [1]. These limitations necessitate adopting scalable, innovative approaches that harness the strengths of advanced sensing technologies.

Remote sensing, particularly through satellite imagery, offers an efficient and scalable solution for biomass estimation. Vegetation indices derived from satellite data, such as the Normalized Difference Vegetation Index (NDVI) and Enhanced Vegetation Index (EVI), are widely used to assess vegetation health and biomass [2,3]. While remote sensing offers a powerful tool for large-scale biomass estimation, inherent limitations exist due to external factors like soil background effects, atmospheric conditions, and sensor noise. [4]. However, these limitations can be effectively mitigated through carefully calibrating and integrating complementary data sources.

Calibration methods, such as those using ground-truth measurements from proximal sensing instruments, help to correct atmospheric and sensor-related distortions, improving the accuracy of remote sensing observations [5]. By implementing appropriate calibration techniques and integrating complementary data, the reliability and accuracy of remote sensing-based biomass estimations can be significantly enhanced.

Proximal sensing technologies complement remote sensing by providing high-resolution, ground-based measurements. Instruments such as the PaddockTrac sonic sensor, which measures pasture height—a critical indicator of biomass—play an essential role in bridging the gap between localized measurements and large-scale satellite observations [6]. These ground-truth measurements are indispensable for calibrating satellite data, thereby enhancing the precision of biomass estimation models. However, proximal sensing alone lacks the capacity to capture broader spatial patterns, underscoring the necessity of integrating it with remote sensing. Integrating proximal and remote sensing data has demonstrated substantial potential to improve model performance, with studies reporting adjusted R^2^ values as high as 0.73 in grassland biomass estimation [7].

The integration of remote and proximal sensing methods creates a robust framework for advanced biomass estimation. Remote sensing offers extensive spatial coverage and systematic monitoring capabilities, while proximal sensing delivers localized, high-precision data. This complementary approach is particularly valuable in heterogeneous landscapes like grasslands and savannas [8], where vegetation structure and composition vary significantly within small areas. Proximal sensors, such as the PaddockTrac sonic sensor, provide detailed ground-level measurements that capture fine-scale variations in pasture height and other biomass indicators [9]. Simultaneously, remote sensing captures landscape-level patterns and trends [10]. Together, these integrated data sources enable a more comprehensive and accurate biomass assessment, accounting for local variations and overall landscape heterogeneity [11].

Machine learning algorithms enhance this integration by effectively handling complex, multi-source data and identifying nonlinear relationships between variables [12,13]. Algorithms such as Random Forest and Extreme Gradient Boosting (XGBoost) have demonstrated considerable success in merging remote and proximal sensing data, significantly reducing error rates and improving prediction accuracy [14,15]. These advanced computational approaches can process diverse inputs—including spectral indices, vegetation height measurements, and meteorological data—to generate more robust and accurate biomass estimates than traditional methods.

This study aims to enhance pasture biomass estimation by integrating proximal sensing measurements from the PaddockTrac ultrasonic sensor with remote sensing data from Landsat and Sentinel satellites. By combining these datasets within a structured analytical workflow and applying advanced machine learning techniques, we seek to develop robust biomass estimation models that capitalize on the complementary strengths of each sensing approach. Specifically, proximal sensors deliver precise, ground-level observations critical for accurate calibration, while satellite imagery offers extensive spatial coverage and scalability, addressing limitations associated with traditional biomass measurement methods.

In addressing existing challenges and methodological gaps in biomass estimation, our research provides practical solutions for improving pasture management sustainability, enabling more informed decision-making and efficient resource allocation in agriculture. The integration of detailed ground-based ultrasonic measurements with satellite-derived vegetation indices, complemented by rigorous machine learning modeling, creates a scalable and precise framework suitable for diverse management scenarios and environmental conditions.

Moreover, this research offers distinct methodological advancements, including the following: (1) a systematic pipeline for integrating high-resolution proximal sensing data with multi-year satellite imagery; (2) an innovative application of the Boruta algorithm for feature selection, effectively identifying influential biophysical predictors from multi-source datasets; and (3) a comparative evaluation of spectral indices, particularly assessing the added value of red-edge vegetation indices. Collectively, these methodological contributions not only address existing limitations in pasture biomass estimation but also provide critical insights and tools for advancing sustainable pasture management and future research.

## 2. Materials and Methods

### 2.1. Data Collection and Study Area

**Figure 1:**
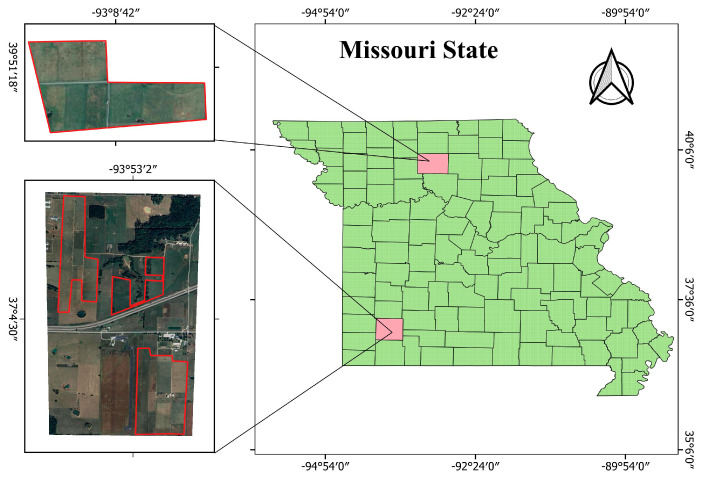
Geographical location and layout of study paddocks in Missouri, USA, used for pasture biomass estimation.

This study analyzed 470 data points collected from five paddocks in Missouri, USA, over six years, beginning in 2011 (Figure 1). The study sites experienced average growing season conditions of 25.8 °C temperature and 23.53 inches of rainfall, significantly influencing vegetation growth and biomass accumulation patterns. Data collection was conducted periodically to capture temporal variations in pasture productivity.

### 2.2. Proximal Sensing Technology

The primary data collection instrument used in this study was the PaddockTrac sensing unit, a novel proximal sensing technology developed by the University of Missouri (MU). This system was employed to collect all the field measurements across the study paddocks throughout the research period.

The PaddockTrac unit employs an ultrasonic sensor at 10 kHz to measure vegetation height above the ground. Based on echo-ranging principles, these sensors emit high-frequency sound waves that reflect off the vegetation canopy, with the travel time of the reflected waves enabling sub-centimeter precision in height calculations (Senix, Hinesburg, VT, USA). Additionally, the sensors capture important parameters, including canopy density and vertical leaf distribution. Canopy density correlates directly with overall plant biomass, while vertical leaf distribution provides critical information on leaf stratification within the canopy, affecting light interception and photosynthetic activity. Both parameters are essential for accurate biomass modeling.

The ultrasonic sensor manufacturer for the PaddockTrac system specifies a measurement accuracy of 2 mm, which was independently verified through a comprehensive two-phase validation protocol. The verification methodology encompassed the following:

1. Controlled Surface Validation: The PaddockTrac system was operated at standard field velocity across a uniform, planar surface (asphalt pavement) to establish baseline calibration parameters and determine zero reference readings under controlled conditions. This procedure enabled the quantification of systematic measurement errors and assessment of instrument precision under idealized field conditions.
2. Field Plot Validation: System performance was evaluated across a diverse set of 200+ experimental plots, where known vegetation heights were measured using a standard ruler as a ground-truth reference [16].

Biomass calibration data for the PaddockTrac system were obtained from field strips immediately harvested following proximal sensing measurements. Specifically, after driving the PaddockTrac unit across a 5 m strip, vegetation from the same area was harvested to a standardized stubble height of 2 cm using a flail-type forage harvester. To ensure representativeness, harvested strips were selected to encompass a broad range of the vegetation heights captured by PaddockTrac, reflecting varying biomass levels. This calibration procedure was conducted at least 25 times per location, repeated every four to six weeks throughout each growing season from 2011 to 2015 [16].

After harvesting, the fresh biomass from each strip was immediately weighed in the field. Subsequently, a representative subsample (approximately 300 g) was collected, weighed, and transported to the laboratory for dry matter determination. These calibration subsamples were dried in a forced-air oven at 55 °C until a stable mass was achieved (typically after 96 h). Each dried subsample was then weighed again to determine its dry matter content accurately.

For geospatial referencing, the PaddockTrac system incorporates a GNSS receiver that records the location of each data point. This geospatial information is systematically cross-referenced with vegetation height measurements, maintaining a precise alignment between spatial and proximal data. This integration enables georeferenced field maps and supports comprehensive spatial analyses for site-specific management planning.

The PaddockTrac hardware is complemented by a mobile application interface and the MU Grazing Wedge web platform (https://grazingwedge.missouri.edu/), which processes raw sensor data using validated height-to-yield equations to convert height measurements into biomass estimates. This integrated approach delivers high-resolution, real-time biomass data essential for effective grazing management decision-making.

All collected proximal sensing data were securely stored and managed on a MySQL server at the University of Missouri, with automated backup protocols implemented to ensure data integrity and availability for analysis.

### 2.3. Remote Sensing Data

Remote sensing parameters were extracted using multispectral satellite imagery processed via Google Earth Engine (GEE). The study utilized Landsat 7 imagery due to its temporal compatibility with calibration cuts from 2011 to 2016. Satellite images were retrieved based on the spatial and temporal coordinates of ground-based data stored in a MySQL database at the University of Missouri. This database served as a central repository for aligning remote sensing data with proximal measurements.

Landsat 7, launched by NASA in 1999, features the Enhanced Thematic Mapper Plus (ETM+) sensor, which captures eight spectral bands critical for Earth surface monitoring. The satellite’s 16-day revisit cycle and near-polar, sun-synchronous orbit provide consistent global coverage, making it a reliable source for vegetation and land cover analysis. Cloud masking and pixel filtering were applied during preprocessing, removing artifacts such as clouds, shadows, and snow to ensure data quality.

Sentinel-2, launched in 2015 by the European Space Agency (ESA) as part of the Copernicus program, comprises two satellites (Sentinel-2A and -2B) that deliver high-resolution multispectral imagery. Equipped with a MultiSpectral Instrument (MSI) (Astrium, Toulouse, France), Sentinel-2 captures data across 13 spectral bands, including 4 red-edge bands designed explicitly for vegetation monitoring. Its 5-day revisit cycle and sun-synchronous, near-polar orbit ensure consistent and frequent global coverage. With spatial resolutions ranging from 10 to 60 m, Sentinel-2 data are highly suitable for detailed land cover classification, assessing vegetation health, and estimating biomass.

Data from Sentinel-2 collected in 2016 were specifically selected for this study, as this year represented the first full operational period of the satellite, providing consistent and high-quality imagery ideal for comparative analysis. Aligning the Sentinel-2 imagery temporally with Landsat 7 data from the same year facilitated a direct and equitable comparison between these two sensors under similar environmental conditions.

The decision to compare biomass estimation models with and without red-edge vegetation indices (VIs) was driven by the need to assess the added value of these spectral bands in predicting biomass accurately. By contrasting models incorporating red-edge information against those excluding it, this study aimed to evaluate the significance of these additional bands for enhancing predictive performance. This analysis is particularly valuable in ecosystems where detecting subtle vegetation stress indicators is essential.

Various vegetation indices were derived from Sentinel-2’s spectral bands to quantify vegetation health, density, and vigor effectively [17]. These indices were selected based on their demonstrated effectiveness in biomass estimation [13,18], their ability to minimize soil background effects [19], and their sensitivity to vegetation stress [20]. Each index was chosen to capture specific vegetation characteristics, ensuring a robust spectral framework for biomass modeling [21,22].

NDVI and SR were incorporated due to their well-established correlations with vegetation health and biomass accumulation [23,24]. MSAVI2 and MSR were specifically chosen to mitigate the influence of soil background, a critical factor in semi-arid regions [25,26]. Additionally, red-edge indices such as NDRE, SRre, and MSRre were utilized to leverage Sentinel-2’s specialized bands for enhanced sensitivity to plant stress and chlorophyll variations. NDWI was also included to evaluate vegetation moisture content, a crucial factor influencing biomass variations due to drought and soil moisture availability [27,28]. Lastly, indices like SR and MSR were favored for their computational simplicity and straightforward interpretation in biomass modeling [29].

The study aimed to provide a comprehensive spectral framework for improving biomass estimation accuracy by integrating these indices. These indices were calculated using established algorithms and standardized workflows to ensure consistency and reproducibility. The VIs employed in this study include the following:

- Normalized Difference Vegetation Index (NDVI):
  (1) $$NDVI=\frac{NIR-RED}{NIR+RED}$$
  NDVI, computed from red and NIR bands, evaluates vegetation density and health. Values range from −1 to +1, where higher values indicate dense, photosynthetically active vegetation, while lower values correspond to sparse or stressed vegetation.
- Green Normalized Difference Vegetation Index (GNDVI):
  (2) $$GNDVI=\frac{NIR-Green}{NIR+Green}$$
  By replacing the red band with the green band, GNDVI reduces the influence of soil reflectance, making it particularly useful in areas with dense vegetation cover.
- Modified Soil-Adjusted Vegetation Index 2 (MSAVI2):
  (3) $$MSAVI2=\frac{2\times NIR+1-\sqrt{{\left(2\times NIR+1\right)}^{2}-8\times (NIR-Red)}}{2}$$
  MSAVI2 incorporates a soil adjustment factor to minimize soil-induced noise, enhancing its utility in arid and semi-arid regions with sparse vegetation.
- Normalized Difference Water Index (NDWI):
  (4) $$NDWI=\frac{Green-NIR}{Green+NIR}$$
  NDWI evaluates water content in vegetation and soil, with higher values indicating moisture-rich areas and lower values identifying arid regions or non-vegetated surfaces.
- Simple Ratio (SR):
  (5) $$SR=\frac{NIR}{Red}$$
  SR is a straightforward measure of vegetation density and health, effective for distinguishing vegetation from non-vegetation surfaces.
- Modified Simple Ratio (MSR):
  (6) $$MSR=\frac{SR-1}{\sqrt{SR+1}}$$
  MSR enhances vegetation signal clarity by reducing soil brightness effects.
- Normalized Difference Red-Edge (NDRE):
  (7) $$NDRE=\frac{NIR-Red\;Edge}{NIR+Red\;Edge}$$
  NDRE leverages red-edge bands to assess chlorophyll content, particularly during mid-to-late growth stages.
- Red-Edge Simple Ratio (SRre) and Modified Simple Ratio Red-Edge (MSRre):
  (8) $$SRre=\frac{NIR}{Red\;Edge}$$
  (9) $$MSRre=\frac{SRre-1}{\sqrt{SRre+1}}$$
  Red-edge vegetation indices (VIs) utilize spectral information from the red-edge region (approximately 690–740 nm) of the electromagnetic spectrum, particularly sensitive to variations in chlorophyll content and plant stress. Sentinel-2 is equipped with dedicated red-edge bands (Bands 5, 6, 7, and 8A), offering enhanced capabilities for vegetation monitoring compared to traditional satellite sensors, such as Landsat 7, which lack these specific spectral bands. Widely adopted red-edge indices, including NDRE and SRre, provide enhanced sensitivity for detecting subtle vegetation stress, enabling the earlier identification of plant health and biomass changes. Additionally, these indices help to reduce interference from atmospheric and soil background effects, further improving the accuracy and reliability of biomass estimation models.

The vegetation indices were integrated with ground-based proximal sensing data, including ultrasonic measurements of vegetation height and GNSS-derived spatial coordinates. Meteorological data were also incorporated to construct robust biomass estimation models. Integration was achieved using a Python-based (v.3.13.2) workflow, synchronizing datasets through spatial and temporal references stored in the MySQL (v.8.4.4) database. Data quality was ensured through preprocessing steps, including the removal of outliers and validation against calibration cuts.

All data, including VIs, satellite imagery, and the Python scripts used for processing, are available upon request for reproducibility. Remote sensing data are accessible through the Google Earth Engine platform, while meteorological variables were sourced from NASA’s Prediction of Worldwide Energy Resources (POWER) database [30].

### 2.4. Weather Data

Accurate meteorological data are critical for understanding environmental processes and their influence on vegetation growth, biomass accumulation, and climate variability. Temperature and precipitation, in particular, are key parameters for modeling vegetation dynamics and biomass estimation. This study utilized the NASA POWER dataset, a comprehensive resource that integrates satellite observations, meteorological reanalysis, and ground-based measurements to provide reliable and consistent climatic data.

The NASA POWER dataset employs sophisticated data processing algorithms to harmonize inputs from various sources, ensuring spatial and temporal consistency across its outputs. Meteorological data are standardized at a 0.5-degree resolution (~55 km) using spatial interpolation techniques and with topographic adjustments refining temperature estimates in areas with elevation variations. Temporal alignment is maintained by resampling data on hourly, daily, and monthly scales, synchronizing timestamps, and applying seasonal corrections to enhance climate trend accuracy. These algorithms include interpolation techniques, quality assurance measures, and the integration of multiple data streams, resulting in a robust dataset suitable for modeling frameworks, decision support systems, and scientific analyses. For this study, temperature and precipitation data were retrieved from NASA POWER via its API, with requests made based on the spatial and temporal coordinates of ground-based measurements stored in a MySQL database.

#### 2.4.1. Integration of Weather Data into Biomass Estimation

Weather data were integrated with proximal and remote sensing datasets to develop a comprehensive framework for biomass estimation. This process combined meteorological parameters, vegetation indices from satellite imagery, and ultrasonic measurements of vegetation height while ensuring accurate spatial and temporal synchronization of data sources.

Spatial synchronization was achieved using the geographical coordinates of the paddocks included in the study. Remote sensing data from Landsat 7 and Sentinel-2 satellites were extracted based on these coordinates, using GEE to precisely align imagery with field measurement locations.

For temporal synchronization, proximal sensing data and satellite imagery were carefully matched according to measurement dates. Proximal measurements were collected on specific dates aligned with field campaigns, while satellite imagery availability varied due to different revisit cycles (16 days for Landsat 7 and 5 days for Sentinel-2). To ensure consistency, the closest cloud-free satellite images available to each field measurement date were selected. Weather data from the NASA POWER database were also temporally aligned by retrieving measurements corresponding precisely to the dates of the field observations. When exact weather records were unavailable, linear interpolation was applied to estimate missing values, thus ensuring continuity and reliability in the combined dataset.

A Python-based script facilitated this integration, leveraging libraries for remote sensing operations, API interactions, and data manipulation. The process followed these steps as follows:

1. Data Preparation: Latitude, longitude, date, and polygon coordinates defining regions of interest were extracted from a MySQL database.
2. Weather Data Retrieval: The NASA POWER API was queried to obtain temperature and precipitation data for each spatial and temporal coordinate.
3. Remote Sensing Data Processing: Landsat 7 and Sentinel-2 imagery was retrieved using the GEE platform. Quality control measures, including cloud and snow masking, ensured high-quality inputs.
4. Vegetation Indices Calculation: Standard formulas were applied to compute VIs such as NDVI, GNDVI, and MSAVI2 from satellite image spectral bands. Mean index values were calculated for each region of interest.
5. Data Integration: Weather data, proximal sensing measurements, and VIs were merged into a unified DataFrame. This integrated dataset enriched the feature set, enhancing its suitability for biomass modeling.

#### 2.4.2. Quality Control and Data Management

Quality control protocols were applied throughout the data integration process. Meteorological data were validated for consistency, while remote sensing inputs were cross-verified against ground-truth calibration cuts. The integrated dataset was securely stored in the MySQL database, ensuring data integrity and accessibility for further analysis.

The integration of weather data with proximal and remote sensing datasets provided a robust platform for analyzing biomass dynamics under varying environmental conditions. The resulting dataset and Python scripts are available upon request, ensuring transparency and reproducibility.

### 2.5. Feature Selection

To enhance model stability and interpretability, feature selection was performed using the Boruta algorithm [31], a wrapper method that identifies the most relevant predictors by iteratively comparing them against randomly shuffled shadow features. Boruta operates alongside Random Forest, evaluating whether a feature consistently contributes more to the model’s predictive power than noise. Unlike conventional feature selection methods, which may overlook weak but relevant predictors, Boruta ensures that all meaningful variables are retained.

To ensure independence among predictors, a multicollinearity assessment was conducted prior to applying the algorithm. A correlation matrix was computed, and features with Pearson correlation coefficients exceeding 0.9 were excluded from the analysis. The refined dataset was then processed using Boruta, which iteratively trained Random Forest models, measured feature importance, and compared each predictor against its shadow counterpart. Features that consistently outperformed shadow features were retained, while irrelevant ones were discarded.

This preprocessing step highlighted key predictors, such as vegetation height, dry matter, and specific vegetation indices (e.g., NDVI and MSAVI2), which were retained for model development. However, Boruta has limitations, particularly in large datasets, where its iterative nature makes it computationally expensive. Additionally, since Boruta is dependent on Random Forest, it may not optimize feature selection for models such as XGBoost or Neural Networks, which rely on different feature importance measures. To mitigate these limitations, an additional SHAP-based analysis was conducted to validate selected features. By addressing multicollinearity, this process reduced redundancy in the dataset and improved the robustness of the machine learning models.

### 2.6. Model Development

To estimate biomass, four machine learning techniques were implemented: Linear Regression, the Decision Tree Regressor, the Random Forest Regressor, and XGBoost (Table 1). Each method was chosen based on its ability to address specific data characteristics, ranging from linear dependencies to complex nonlinear relationships. These models were applied to an integrated dataset containing vegetation indices, proximal sensing data, and weather parameters.

Linear Regression served as a baseline model due to its simplicity and interpretability. The model predicted biomass as a linear function of predictor variables, expressed mathematically as $y=\beta_{0}+\beta_{1}x+\epsilon$, where *y* is biomass, *x* is the predictor variable, $\beta_{0}$ is the intercept, $\beta_{1}\;$is the slope, and *ϵ* represents the error term. Despite its ease of interpretation, Linear Regression is inherently limited by its assumption of linearity, restricting its ability to capture complex nonlinear relationships commonly observed in pasture biomass data [32]. Therefore, more advanced models capable of capturing nonlinear dynamics were required for accurate biomass estimation [18].

The Decision Tree Regressor, a non-parametric technique, was employed to model nonlinear interactions by recursively splitting data into subsets that minimize variance. The resulting tree structure captured complex relationships between vegetation indices, weather conditions, and biomass that Linear Regression would otherwise overlook. However, it is prone to overfitting, especially in datasets with high variability. The Random Forest Regressor, an ensemble method, was introduced to mitigate this limitation.

Random Forest is an ensemble method that builds multiple Decision Trees on random subsets of data and aggregates their predictions to improve accuracy and generalization. It also provides feature importance metrics, aiding in understanding the contribution of each predictor. This reduces sensitivity to noise by averaging multiple trees.

We utilized XGBoost, a powerful gradient-boosting algorithm renowned for its predictive accuracy and capacity to capture complex nonlinear relationships, to estimate pasture biomass. By iteratively optimizing model performance through gradient boosting, XGBoost systematically reduces prediction errors, while its robust regularization methods prevent overfitting, making it particularly effective in vegetation monitoring scenarios.

To further enhance model generalization and reliability across diverse environmental conditions, we employed a stratified cross-validation strategy designed to retain the temporal and spatial integrity of our dataset during training. This careful approach ensured that the model accurately identified meaningful biophysical relationships rather than simply statistical correlations. Our implementation thus built upon existing machine-learning frameworks, refining the application of XGBoost through targeted optimizations tailored specifically for agricultural contexts.

### 2.7. Model Training and Evaluation

The dataset for this study included vegetation height, dry matter, various vegetation indices (e.g., mean NDVI and mean NDWI), temperature, precipitation, Julian date, and year as predictors. The data were split into training (80%) and testing (20%) subsets to ensure robust evaluation. This approach allowed the models to learn from most of the data while reserving an independent subset for performance validation. The training data were used for model development and hyperparameter tuning, while the testing subset served as an independent validation set to assess predictive performance. Before splitting, the dataset was randomly shuffled to prevent any inherent ordering bias (e.g., temporal or spatial patterns).

Predictors were standardized to have a mean of 0 and a standard deviation of 1, a critical preprocessing step for algorithms sensitive to feature scaling, such as Linear Regression and XGBoost. Standardization ensures balanced feature contributions, preventing biases and enhancing model stability and performance. It is a simple yet effective step to improve the robustness and accuracy of machine learning workflows.

To enhance model stability and generalizability, a five-fold cross-validation strategy was employed. This approach divided the training data into five subsets, each serving as a validation set once. This yielded average R^2^ scores and standard deviations, providing a reliable assessment of model performance across multiple iterations. Once trained and optimized using cross-validation, the final model was evaluated on the test set to measure generalization performance.

Model performance was assessed through several key metrics. The coefficient of determination (R^2^) quantified the proportion of variance in biomass explained by each model. The Root Mean Square Error (RMSE) highlighted larger discrepancies between the observed and predicted values, while the Mean Absolute Error (MAE) captured the average magnitude of errors. The Mean Bias Error (MBE) further provided insights into systematic over- or under-predictions.

Scatter plots and residual plots were analyzed to visualize prediction accuracy and error distributions to complement these quantitative evaluations. Scatter plots illustrated the alignment between the predicted and observed biomass values, while residual plots revealed potential biases and systematic patterns in the residuals. Together, these analyses ensured a rigorous evaluation of model performance and informed the selection of the most reliable and accurate approach to biomass prediction.

## 3. Results

### Dataset Performance

The performance of four machine learning models was evaluated using Landsat 7 and Sentinel-2 datasets across different scenarios. These scenarios included Landsat 7 spanning all available years, Landsat 7 limited to 2016, Sentinel-2 incorporating red-edge VIs for 2016, and Sentinel-2 without red-edge VIs for 2016. Among all scenarios and datasets, XGBoost consistently delivered the highest predictive accuracy and robustness for biomass estimation.

When analyzing Landsat 7 data across all years without red-edge VIs, XGBoost demonstrated superior performance. It achieved an R^2^ of 0.86, an MAE of 414 kg/ha, an RMSE of 538 kg/ha, and an MBE of −43 kg/ha (Table 2). These errors correspond to 12% (MAE) and 15% (RMSE) of the mean observed biomass during the growing season, underscoring the model’s exceptional accuracy. Random Forest was the second-best model, with an R^2^ of 0.82, while the Decision Tree and Linear Regression models demonstrated lower R^2^ values of 0.70 and 0.60, respectively.

**Figure 2:**
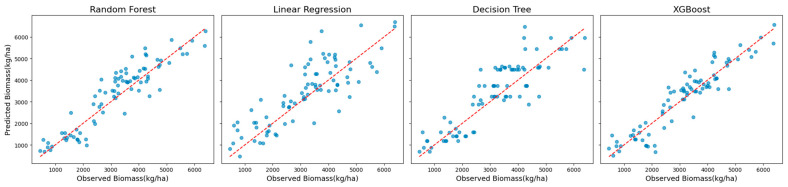
Scatter plots of observed vs. predicted biomass values for all models using Landsat 7 data across all years (excluding red-edge VIs).

**Figure 3:**
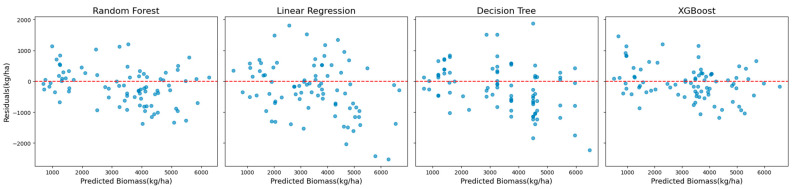
Residual plots showing the distribution of prediction errors for the models using Landsat 7 data across all years (excluding red-edge VIs).

The superior performance of XGBoost is further illustrated in Figure 2, which shows scatter plots of the observed versus predicted biomass values. These plots reveal a tight clustering of points along the diagonal, indicating strong alignment between predictions and observations. The residual plots presented in Figure 3 complement this analysis by showing well-distributed residuals with minimal variance and no noticeable systematic biases.

This comparative analysis highlights XGBoost’s advanced capability to model complex nonlinear relationships while mitigating overfitting through regularization techniques. Its robustness and accuracy across diverse scenarios establish XGBoost as a reliable method for biomass estimation, effectively handling varying datasets and challenging modeling conditions.

The analysis of feature importance identified “Height”, “Dry Matter”, “Mean NDVI”, “Temperature”, “Precipitation”, “Julian Date”, and “Year” as the most influential predictors across all models. In the analysis, feature importance identified temperature and precipitation as influential predictors, suggesting that variations in these factors have a measurable impact on biomass. This aligns with domain knowledge, where temperature and precipitation change affect crop development, photosynthesis rates, and overall yield. Their inclusion ensures that the model accounts for environmental variability, improving the accuracy and reliability of biomass predictions.

For Sentinel-2 data incorporating red-edge VIs, XGBoost again delivered the best performance, achieving an R^2^ of 0.79, an MAE of 464 kg/ha, an RMSE of 716 kg/ha, and an MBE of 132 kg/ha (Table 3). The MAE, equivalent to 15% of the mean observed biomass, underscored the model’s suitability for practical applications. While XGBoost exhibited a slight positive bias (MBE = 132 kg/ha), this was only marginally higher than that for Random Forest (MBE = 116 kg/ha). The Random Forest and Decision Tree models produced similar but slightly lower performance, while Linear Regression had the lowest predictive power with an R^2^ of 0.67.

**Figure 4:**
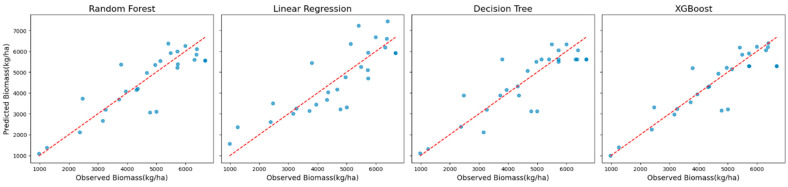
Scatter plots of observed vs. predicted biomass values for all models using Sentinel-2 data with red-edge VIs.

Scatter plots of observed versus predicted biomass values (Figure 4) were used to examine model behavior. These plots showed that XGBoost predictions aligned most closely with the observed values across the biomass range, whereas Random Forest predictions exhibited a slight dispersion at higher biomass levels.

**Figure 5:**
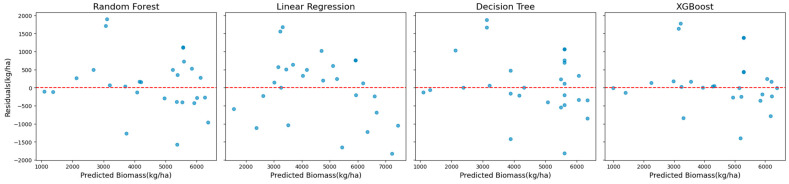
Residual plots showing distributions of prediction errors for models using Sentinel-2 data with red-edge VIs.

Residual plots (Figure 5) provided additional insights into systematic biases. XGBoost and Random Forest demonstrated minimal residual variance, with residuals tightly centered around zero, indicating reliable and robust model performance.

The analysis of Landsat 7 data limited to 2016 produced consistent results, with XGBoost maintaining its position as the best-performing model. It achieved an R^2^ of 0.79, an MAE of 462 kg/ha, and an RMSE of 712 kg/ha (Table 4). Other models, including Random Forest and Decision Tree, followed closely, while Linear Regression exhibited the weakest performance, with an R^2^ of 0.65. Regarding bias, XGBoost showed a small positive MBE of 152.47 kg/ha, indicating slight over-prediction. In contrast, Linear Regression had a near-zero MBE, but its overall poor predictive performance made it a less competitive option.

**Figure 6:**
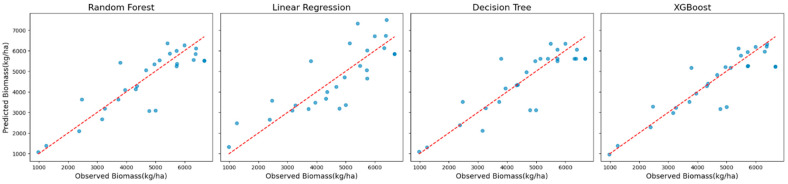
Scatter plots of observed vs. predicted biomass values for all models using Landsat 7 data for 2016 only (excluding red-edge VIs).

**Figure 7:**
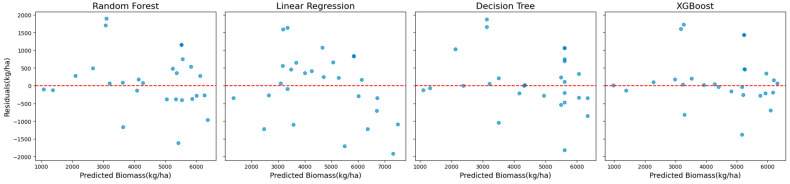
Residual plots showing distributions of prediction errors for models using Landsat 7 data for 2016 only (excluding red-edge VIs).

The differences in model behavior were further illustrated by scatter and residual plots. Scatter plots (Figure 6) showed that XGBoost and Random Forest predictions were tightly clustered along the diagonal, indicating high accuracy. Residual plots (Figure 7) confirmed minimal systematic bias for these models, further supporting their reliability for practical applications.

Overall, XGBoost emerged as the most accurate and reliable model for biomass estimation. While Random Forest presented a reasonable alternative, it fell short of XGBoost regarding error metrics and bias, solidifying XGBoost’s position as the preferred choice for this analysis.

**Figure 8:**
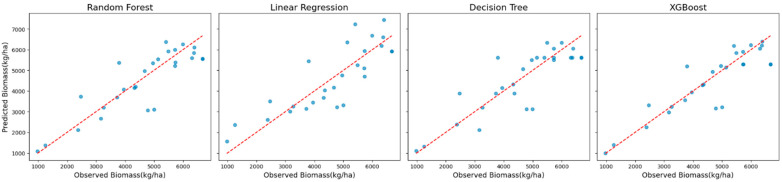
Scatter plots of observed vs. predicted biomass values for all models using Sentinel-2 data without red-edge VIs.

The performance metrics for Sentinel-2 data excluding red-edge VIs were comparable to those obtained when red-edge VIs were included. XGBoost maintained its superior performance, achieving an R^2^ of 0.79, an MAE of 464 kg/ha, and an RMSE of 716 kg/ha. These values correspond to 15% and 20% of the mean observed biomass, respectively, indicating high predictive accuracy (Figure 8). Random Forest and Decision Tree followed, with slightly lower performance metrics (Table 5).

The MBE was calculated for all the models to evaluate potential biases. XGBoost exhibited a small positive bias (MBE = 132 kg/ha), indicating a slight tendency to over-predict. Linear Regression, in contrast, had a negligible bias (MBE = −8 kg/ha); however, its poor predictive capability, as reflected in its higher MAE and RMSE values, limited its utility for biomass estimation.

**Figure 9:**
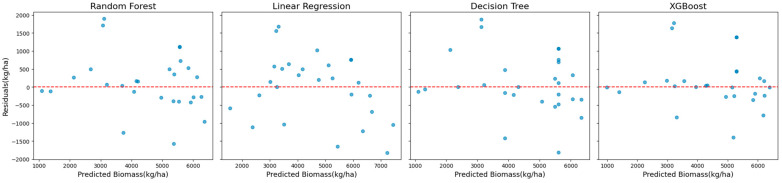
Residual plots showing distributions of prediction errors for models using Sentinel-2 data without red-edge Vis.

Residual plots (Figure 9) provided further insights into error distributions. XGBoost and Random Forest displayed minimal systematic biases, with residuals tightly clustered around zero, reinforcing their reliability. Decision Tree, however, showed greater variability in residuals, particularly for higher predicted values, reducing its reliability for extreme cases. Although Linear Regression demonstrated relatively consistent residuals, its inability to capture the nonlinear relationships inherent in the data resulted in inferior overall performance.

In summary, XGBoost remained the most accurate and reliable model for Sentinel-2 data, excluding red-edge VIs, with Random Forest as a viable alternative despite slightly higher error rates.

Feature importance analysis using the Boruta algorithm identified “Height”, “Dry Matter”, and “mean MSAVI2” as the most influential predictors of biomass. The importance of these predictors was interpreted based on their role in biomass estimation. These findings were analyzed based on their biophysical significance and alignment with established domain knowledge, confirming the model’s predictive capability and interpretability.

## 4. Discussion

This study underscores the importance of integrating high-quality ground-truth data with remote sensing and advanced machine-learning techniques for accurate pasture biomass estimation. Our framework effectively bridges localized field observations and large-scale remote sensing data by combining precise vegetation height measurements obtained from the PaddockTrac proximal sensing system with satellite-derived vegetation indices. These results reinforce previous findings emphasizing the critical role of accurate in situ data for calibrating and validating remote sensing models [33].

Feature importance analysis identified vegetation height, dry matter content, and mean MSAVI2 as the most influential predictors of biomass. The prominence of vegetation height as a predictor aligns with its established role as a structural indicator directly linked to biomass accumulation [34], reflecting conditions favorable for growth, such as adequate nutrients and moisture availability. Dry matter content directly measures plant productivity and is a well-established proxy for biomass estimation [18]. Among the spectral indices, mean MSAVI2 emerged as particularly effective, largely due to its ability to mitigate soil background interference—an important consideration in heterogeneous pasture ecosystems with variable soil exposure [26].

The strength and interpretability of these predictors contributed significantly to the model’s high predictive accuracy. The clear biophysical relevance of these variables, combined with their consistent alignment with established domain knowledge, underscores the practical applicability and robustness of the proposed model for pasture management decision-making.

### 4.1. The Value of Proximal Sensing in Biomass Estimation

Reliable ground-truth data are indispensable for remote sensing-based biomass models, particularly in grassland ecosystems characterized by pronounced spatial and temporal variability. Proximal sensing systems like PaddockTrac offer detailed, high-resolution, site-specific measurements essential for calibrating vegetation indices and machine learning models. The sub-centimeter accuracy and integrated GNSS capabilities of PaddockTrac facilitate the precise calibration of vegetation indices sensitive to vegetation health, such as NDVI and MSAVI2. These findings align closely with recent studies demonstrating the utility of high-precision proximal sensing technologies to bridge gaps between field observations and satellite imagery [35].

Compared with conventional methods, PaddockTrac offers significant advantages. For example, the Rising Plate Meter (RPM), a commonly used tool, achieved RMSE values ranging from 226 to 347 kg/ha when calibrated with satellite-derived NDVI [5]. However, these methods often require frequent recalibration to account for pasture composition and grazing intensity variations, limiting their scalability. In contrast, integrating PaddockTrac with remote sensing data achieved RMSE values of 538 to 716 kg/ha, demonstrating its capacity to address complex environments accurately.

When evaluating biomass estimation approaches, destructive sampling remains a reliable gold standard but is often prohibitively expensive and time-consuming for large areas. Meanwhile, devices like the Rising Plate Meter require frequent recalibration and yield less spatial detail, compromising their cost-effectiveness over extensive landscapes. PaddockTrac addresses these limitations by integrating sub-centimeter height precision with automated GPS tracking, allowing users to measure pasture biomass rapidly across multiple paddocks with minimal labor. This high-throughput approach reduces operational costs while maintaining robust accuracy, making PaddockTrac particularly advantageous for frequent monitoring of large-scale grazing systems.

The challenges of scaling biomass estimation to larger, more variable landscapes are evident when comparing results across studies. Zhang et al. [36] achieved lower RMSEs (91 kg/ha) in controlled small-scale plots using unmanned aerial vehicle (UAV) systems, highlighting the increased difficulty of maintaining similar accuracies over extensive, heterogeneous areas, as examined in our study.

### 4.2. Machine Learning Models and Dataset Comparisons

In this study, XGBoost outperformed other machine learning algorithms, including Random Forest, Decision Trees, and Linear Regression, across all tested scenarios. This finding aligns with Defalque et al. [18], who similarly noted XGBoost’s advantage over Random Forest models in predictive tasks. The algorithm’s consistent superiority stems from its advanced design, which offers several key strengths tailored to the complexities of pasture biomass estimation.

XGBoost’s exceptional performance is driven by its iterative gradient-boosting framework. Unlike single-pass models, this approach systematically refines predictions by addressing residual errors from prior iterations, making it particularly adept at capturing the intricate patterns in pasture biomass data. The algorithm’s tree-based structure further enhances its ability to model nonlinear relationships among diverse inputs such as vegetation indices (e.g., NDVI, MSAVI2), vegetation height from PaddockTrac, and environmental factors like temperature and precipitation. Additionally, XGBoost incorporates robust regularization mechanisms that prevent overfitting, a critical feature for handling the high seasonal variability and heterogeneity inherent in pasture systems. By effectively integrating these multi-source datasets, XGBoost exhibited minimal systematic prediction bias across all the experimental setups, echoing findings by Freitas et al. [25] in their above-ground biomass studies.

These results both reinforce and expand upon prior environmental monitoring research. Chen and Guestrin [15] established XGBoost’s computational efficiency and predictive power across various domains, while Yang et al. [37] highlighted its effectiveness for grassland biomass estimation in the Three-River Headwaters Region. Our study extends these insights by demonstrating XGBoost’s versatility across different satellite platforms (Landsat 7 and Sentinel-2) and spectral configurations. This cross-platform robustness underscores its potential as a reliable tool for operational pasture monitoring systems that leverage diverse remote sensing data sources. Our findings align with multi-platform approaches described by Dos Reis et al. [38] and are enriched by Andersson et al. [39], who explored the interplay between pasture biomass, height, and vegetation indices, providing additional context for XGBoost’s success in this context.

In this study, XGBoost achieved an RMSE of 538 kg/ha using Landsat data, outperforming the other tested approaches. This performance is consistent with the results of Alckmin et al. [21], who reported RMSE values of approximately 397 kg/ha utilizing multispectral UAV imagery combined with Cubist regression. The enhanced accuracy in their study likely reflects the advantages of rigorous radiometric calibration and the Cubist algorithm’s design, which effectively balances prediction accuracy and interpretability.

Chen et al. [12] also achieved RMSE values of 356 kg/ha using neural networks, but their inclusion of climate variables alongside Sentinel-2 imagery may have enhanced accuracy. In contrast, this study relied solely on remote and proximal sensing inputs, demonstrating comparable performance while maintaining a more straightforward modeling framework.

Otgonbayar et al. [40] achieved lower RMSE values, ranging from 98 to 101 kg/ha, in studies conducted in less complex environments using Partial Least Squares Regression. Similarly, Pranga et al. [41] demonstrated the effectiveness of combining UAV-based structural and spectral data, achieving RMSE values between 345 and 400 kg/ha for perennial ryegrass biomass estimation. These findings underscore the importance of integrating structural features, such as canopy height, with spectral data to capture variability in vegetation better.

Interestingly, our study revealed no substantial differences in biomass prediction accuracy between the Landsat 7 and Sentinel-2 datasets, even when incorporating Sentinel-2’s red-edge VIs. The lack of improvement from Sentinel-2’s red-edge VIs can likely be attributed to the specific characteristics of our study area. The relatively homogeneous vegetation cover meant that conventional broad-band indices such as MSAVI2 and NDVI already captured the essential spectral information related to biomass. In such environments, these established indices perform robustly, mainly where soil background effects are significant [42].

Red-edge indices typically demonstrate their value in more complex scenarios—specifically in heterogeneous canopies or vegetation experiencing stress conditions [43]. In these situations, they excel at detecting subtle variations in chlorophyll content that traditional indices might miss. For instance, narrow-band red-edge VIs have effectively addressed saturation issues at high biomass levels that commonly affect broader band indices like NDVI [44].

However, the minimal vegetation stress and low variability in our study area likely created spectral redundancy between the red edge and other spectral bands. This redundancy effectively diminished any additional value that red-edge indices could offer for biomass estimation in this particular context.

This observation aligns with broader research findings indicating that while red-edge indices show promise in improving biomass predictions in heterogeneous or semi-arid regions with higher vegetation stress and variability [45], their advantages may not materialize in more uniform vegetation conditions. Further investigation into the specific environmental conditions and vegetation characteristics of our study area could provide additional insights into this phenomenon.

### 4.3. Implications for Precision Agriculture and Rangeland Management

Integrating proximal and remote sensing data provides a reliable framework for advancing precision agriculture and rangeland management. The RMSE values achieved in this study are within acceptable ranges for operational applications, supporting tasks such as grazing optimization, forage availability monitoring, and carbon stock assessments. Similar approaches, such as the integration of multispectral and radar data in semi-arid grasslands, have also been shown to enhance biomass estimation accuracy [46].

Moreover, XGBoost’s demonstrated effectiveness positions it as a valuable tool for operationalizing large-scale biomass monitoring. The proposed framework addresses the critical challenge of balancing spatial coverage with measurement accuracy by combining robust predictive models with high-quality ground-truth data. This capability is particularly relevant for applications requiring frequent updates, such as adaptive grazing management and climate resilience planning [35].

### 4.4. Methodological Contributions

The integrated sensing approach developed in this study contributes to the field of pasture monitoring and biomass estimation by combining proximal and remote sensing data sources. While previous studies have utilized either proximal or remote sensing data separately, our approach integrates these complementary data sources to address certain limitations inherent to each method when used independently.

When comparing our results with previous work, we note that traditional vegetation index-based approaches typically achieve R^2^ values between 0.45 and 0.65 for pasture biomass estimation [42,45]. Our machine learning framework achieved an R^2^ value of 0.86 with an RMSE of 538 kg/ha, suggesting that the integrated approach may offer improvements in certain contexts.

A notable aspect of our approach is the feature selection methodology using the Boruta algorithm with consideration of biophysical constraints. This approach aims to ensure that selected features maintain ecological relevance while also providing statistical significance, addressing a common challenge in machine learning applications for agricultural monitoring—balancing statistical performance with biophysical interpretability.

Our comparative assessment of spectral configurations, particularly regarding red-edge indices, provides additional insights into their utility across different vegetation contexts. While the literature often suggests the benefits of red-edge indices for vegetation monitoring [43,44], our evaluation indicates that their contribution may vary with vegetation homogeneity and stress conditions. This finding may inform sensor selection and data processing decisions in pasture monitoring applications.

The methodological approach described here may be adaptable to various agricultural environments, potentially contributing to the development of biomass estimation tools for land management applications.

### 4.5. Limitations and Research Opportunities

While the proposed framework demonstrates significant advancements, it highlights areas requiring further development. Ultrasonic sensors, as employed by the PaddockTrac system, can experience biases under dense vegetation or sparse cover conditions, where signal interference might reduce measurement accuracy. To address this, future work should consider integrating complementary sensing technologies such as LiDAR or hyperspectral imaging, which offer enhanced capabilities in capturing canopy structure and vegetation health [47].

Scalability is another constraint of proximal sensing. Further technological advancements in automation, miniaturization, and integration with UAVs or IoT frameworks are essential for broader applications across larger landscapes [48]. Moreover, although red-edge indices provided limited improvements in our specific scenario, exploring their utility in more heterogeneous or stressed environments could yield valuable insights.

### 4.6. Future Research

Building on the insights from this study, future research should focus on the following:

- Expanding the capabilities of proximal sensing systems, including measurements of canopy density, leaf area index, and below-ground biomass.
- Leveraging high-resolution remote sensing platforms like PlanetScope for more detailed spatial and temporal monitoring.
- Exploring advanced machine learning models, including deep learning architectures, to improve multi-source data integration.
- Validating the framework across diverse ecosystems to ensure its scalability and adaptability under varying environmental conditions.

Future research can further refine biomass estimation frameworks by addressing these challenges, contributing to sustainable agricultural practices and improved ecosystem management.

## 5. Conclusions

This study presents an integrated approach to pasture biomass estimation that effectively combines proximal sensing, remote sensing, and machine learning techniques. By integrating PaddockTrac ultrasonic measurements for precise vegetation height determination with satellite-derived vegetation indices, we have addressed key limitations in traditional biomass estimation methods. Our results demonstrate that this combined approach offers improved prediction accuracy while maintaining ecological interpretability.

Among the machine learning methods evaluated, XGBoost consistently delivered the most robust predictions, highlighting the potential of advanced algorithms to enhance biomass estimation in agricultural contexts. Importantly, our findings confirm that freely available satellite data from Landsat 7 and Sentinel-2 provide sufficient spatial resolution and temporal frequency to support practical pasture monitoring and precision agriculture applications. This integrated framework establishes a foundation for more effective and scalable biomass estimation tools that can support sustainable grassland management practices.

Despite its strengths, the study also revealed limitations. While PaddockTrac significantly reduces the time required to assess biomass compared to walking each pasture, driving the ATV unit over each pasture still requires time and effort that may be impractical for producers, especially on farms larger than 200 hectares. Addressing this challenge will require more scalable solutions, such as automated ground-based robotic units or integrating the sensors into UAVs for fully automated data collection. Additionally, although red-edge indices did not substantially improve biomass predictions in this relatively homogeneous setting, they may still prove beneficial in more heterogeneous or stressed environments.

From a practical standpoint, the integrated framework offers several immediate applications in farm management and precision agriculture, including optimized grazing schedules, improved forage budgeting, and refined carbon stock assessments. These outcomes not only enhance day-to-day operational decisions but also support longer-term sustainability goals for ranchers and land managers. Going forward, further improvements could include adding sensors capable of measuring density and other canopy attributes to the proximal sensing system, incorporating higher-resolution imagery (e.g., PlanetScope), and exploring deep learning methods for complex feature extraction. Extending this work to larger or more heterogeneous landscapes could broaden the framework’s utility to entire farms, watersheds, or even ecosystems, especially where detailed biomass data are critical for productivity and environmental stewardship.

In summary, this study underscores the value of high-quality ground-truth measurements in calibrating and validating remote sensing data for biomass estimation, demonstrating how well-designed machine learning models can capitalize on these integrated datasets. Future research should continue refining these techniques to support more scalable, automated, and comprehensive solutions, ultimately contributing to the global pursuit of sustainable agriculture and effective land management.

## Figures and Tables

**Table 1:** Characteristics and performance attributes of machine learning regression models for pasture biomass estimation.

Model	Type	Strengths	Weaknesses	Overfitting Control
Linear Regression	Parametric	- Simple - Interpretable	- Assumes linear relationships between variables	N/A
Decision Tree Regressor	Non-parametric	- Captures nonlinear interactions - Easy to visualize	- Prone to overfitting	- Max depth - Pruning
Random Forest Regressor	Ensemble (Bagging)	- Reduces overfitting - Provides robust feature importance rankings	- Less interpretable - Computationally intensive	- Bootstrapping - Averaging outputs
XGBoost	Ensemble (Boosting)	- High accuracy - Handles complex interactions, regularization	- Computational cost - Hyperparameter tuning required	- Regularization - Early stopping

**Table 2:** Model performance metrics using Landsat 7 data across all years (excluding red-edge VIs).

Model ^1^	Test R^2^	MAE (kg/ha)	RMSE (kg/ha)	MBE (kg/ha)
Linear Regression	0.60	700	895	−250
Decision Tree	0.70	610	773	−207
Random Forest	0.82	474	599	−158
XGBoost	**0.86**	**414**	**538**	**−43**

^1^ Selected features: height, dry matter, mean NDVI, temperature, precipitation, Julian date, year.

**Table 3:** Model performance metrics using Sentinel-2 data with red-edge VIs.

Model ^1^	Test R^2^	MAE (kg/ha)	RMSE (kg/ha)	MBE (kg/ha)
Linear Regression	0.67	721	887	**−9**
Decision Tree	0.72	605	821	88
Random Forest	0.75	578	779	116
XGBoost	**0.79**	**464**	**716**	131

^1^ Selected features: height, dry matter, mean MSAVI2.

**Table 4:** Model performance metrics using Landsat 7 data for 2016 only (excluding red-edge VIs).

Model ^1^	Test R^2^	MAE (kg/ha)	RMSE (kg/ha)	MBE (kg/ha)
Linear Regression	0.65	745	913	**−20**
Decision Tree	0.74	571	792	105
Random Forest	0.75	581	783	123
XGBoost	**0.79**	**462**	**712**	152

^1^ Selected features: height, dry matter, mean NDVI.

**Table 5:** Model performance metrics using Sentinel-2 data without red-edge VIs.

Model ^1^	Test R^2^	MAE (kg/ha)	RMSE (kg/ha)	MBE (kg/ha)
Linear Regression	0.67	721	887	**−8**
Decision Tree	0.72	605	821	88
Random Forest	0.75	578	779	116
XGBoost	**0.79**	**464**	**716**	132

^1^ Selected features: height, dry matter, mean MSAVI2.

## Data Availability

Data are contained within the article.
